# DOACs in Patients With Giant Coronary Artery Aneurysms After Kawasaki Disease

**DOI:** 10.1001/jamanetworkopen.2023.43801

**Published:** 2023-11-10

**Authors:** Kirsten B. Dummer, Koichi Miyata, Chisato Shimizu, Adriana H. Tremoulet, Jill Gleason, John B. Gordon, Jane C. Burns

**Affiliations:** 1 Department of Pediatrics, University of California San Diego, Rady Children’s Hospital, San Diego; 2 San Diego Cardiac Center, San Diego, California

## Abstract

This case series examines outcomes among patients with giant coronary artery aneurysms after Kawasaki disease treated with direct oral anticoagulants (DOACs).

## Introduction

Coronary artery aneurysms (CAA) result from intense inflammation during acute Kawasaki disease (KD), with resultant destruction of the normal architecture of the vessel wall. The altered rheodynamics through the diseased artery result in decreased wall shear stress and increased particle residence time, 2 major risk factors for ischemia and thrombosis.^1^ Although to our knowledge, no randomized clinical trials exist to guide management, there is consensus among international guidelines that patients with aneurysms measuring 8 mm or *Z*-score (internal diameter normalized for body surface area) of 10 SD units or greater should be treated with systemic anticoagulation and antiplatelet therapy.^2,3^ Warfarin and low–molecular weight heparin (LMWH) are the standard anticoagulants for these patients, but each have significant drawbacks. Limitations of warfarin include a narrow therapeutic range, food and medication interactions, need for frequent monitoring with phlebotomy or fingerstick, bleeding complications, and vascular wall calcification due to inhibition of the enzyme matrix γ-carboxyglutamate Gla protein that scavenges calcium phosphate in the tissues.^4^ Therapeutic low–molecular weight heparin requires twice daily subcutaneous injections that cause discomfort and can affect quality of life in children. Therefore, we offered direct oral anticoagulants (DOACs) to a group of patients with KD and giant CAA as an option for systemic anticoagulation.

## Methods

This is a case series of 24 patients with KD who required systemic anticoagulation due to giant CAA or regional hypokinesis secondary to thrombotic sequelae of KD and opted to be treated with a DOAC. The institutional review board of University of California, San Diego, approved this study, and consent or assent for longitudinal studies were obtained. This study is reported following the Strengthening the Reporting of Observational Studies in Epidemiology (STROBE) reporting guideline. Records from the adult and pediatric KD clinics were reviewed (San Diego Cardiac Center, Rady Children’s Hospital San Diego, California). Patients were followed-up for 1.9 to 30 years.

## Results

A total of 24 patients with KD (median [IQR] age, 33.8 [22.7-49.2] years; 19 male) were treated with DOACs for systemic anticoagulation. Eight patients received intravenous immunoglobulin and/or aspirin at the time of acute presentation, 6 patients received delayed intravenous immunoglobulin treatment, and 10 patients were never recognized or treated until presentation in adulthood with myocardial infarction or symptoms requiring catheterization. The median (IQR) observation period with DOAC therapy was 4.9 (4.2-8.0) years, with 138 total treatment-years observed (Table). Of 24 patients, 6 started a DOAC at younger than 18 years. There were no major bleeding events during the observation period. Major adverse cardiac events occurred in 3 patients: 1 patient treated with dabigatran (patient No. 9) experienced myocardial infarction with left anterior descending occlusion while engaged in strenuous exercise (Table), 1 patient using rivaroxaban (patient No. 10) experienced an ischemic event with a filling defect in both branches of the obtuse marginal off the circumflex due to distal thrombus, and 1 patient using apixaban (patient No. 13) experienced recurrent myocardial infarction.

**Table.** Demographic and Clinical Characteristics of the Study Population

Patient No.	Age at KD onset, y	Sex	DOAC duration, y	Age at DOAC initiation, y	DOAC	CAA dimensions, mm (*Z*-score)^a^		CT calcium score, mm^3^	KD diagnosis and treatment
						LAD	RCA		
1	2	Male	4.2	4	Apixaban	9.1 (21.5)	7.5 (15)	ND	Treated with IVIG, IFX, and atorvastatin
2	5	Male	5.8	8	Apixaban	9.9 (18)	8.4 (14.2)	ND; calcifications noted in RCA	Treated with IVIG, IFX, and atorvastatin
3	8	Male	5.0	8	Apixaban	5 (5.3)	8.2 (11.8)	ND	Treated with IVIG, IFX, CSA, and atorvastatin
4	2-3	Male	8.8	13	Apixaban	5-6 (5)	10 (14)	142; age 17 y^b^	Delayed diagnosis on day 12, treated with IVIG
5	6	Male	4.5	13	Apixaban	7 (14.8)	Proximal, 4.8 (6.9); distal, 12.5 (ND)	0; age 16 y^b^	Treated with IVIG and steroids
6	16	Male	8.3	16	Apixaban	6.8 (8.2)	7.5 (8.1)	231; age 24 y	Delayed diagnosis 3 wk from fever onset, treated with IVIG and IFX
7	1	Female	2.6	18	Apixaban	18 (ND)	14 (ND)	ND; calcifications noted in LAD and RCA^b^	Treated with IVIG twice
8	13^c^	Male	4.3	19	Apixaban	9 (ND)	7-8 (ND)	872; age 19 y	Missed diagnosis until MI at age 19 y
9	2^c^	Male	11.9	20	Dabigatran	9 (ND); Cx, 9	11 (ND)	0; age 22 y	Missed diagnosis until MI at age 20 y
10	0.8	Male	4.3	24	Rivaroxaban	4.5 (10); Cx, 3.2 (5.8)	4.3 (8)	0; age 24 y^b^	Delayed diagnosis 3 wk from fever onset, treated with IVIG twice
11	1.5	Male	6.4	25	Apixaban	8 (11.7)	5 (3.6)	250; age 25 y^b^	Treated with IVIG
12	0.3	Female	4.7	26	Apixaban	9 (ND); LMCA, 14	9.5 (ND)	ND	Delayed diagnosis 3 wk from fever onset, treated with IVIG
13	ND	Female	7.1	28	Apixaban	ND	ND	ND	Missed diagnosis until MI at age 28 y
14	ND	Male	4.8	34	Apixaban	22 (ND)	53 (ND)	8677; age 34 y^b^	Missed diagnosis until MI at age 22 y
15	7	Female	7.1	36	Apixaban	9.5 (ND)	11 (ND)	2421; age 36 y^b^	Delayed diagnosis several wk after fever onset, treated with IVIG
16	1-2	Male	8.7	37	Rivaroxaban	ND; Cx, 7 (NA)	4 (ND)	2037; age 42 y	Treated with aspirin only
17	15	Male	4.2	41	Rivaroxaban	11 (ND); LMCA, 14	8 (ND)	ND	Delayed diagnosis 4 wk after fever onset, treated with aspirin only
18	16	Female	1.9	43	Apixaban	10 (ND)	8 (ND)	1074; age 44 y^b^	Treated with IVIG
19	ND	Male	3.6	46	Apixaban	ND	Occlusion of giant aneurysm	ND	Missed diagnosis until MI at age 34 y
20	ND	Male	6.4	48	Rivaroxaban	6 (ND)	8 (ND)	ND	Missed diagnosis until MI at age 46 y
21	5^c^	Male	3.1	56	Apixaban	Proximal dilatation; Cx, 8	Dilatation with irregularity	ND	Missed diagnosis until MI at age 45 y
22	ND	Male	8.3	58	Rivaroxaban	ND	8 (ND)	ND	Missed diagnosis until cardiac catheterization at age 58 y
23	8-9	Male	8.6	59	Rivaroxaban	Proximal aneurysm	Diffuse aneurysm	0; age 55 y^b^	Missed diagnosis until MI at age 54 y
24	6^c^	Male	3.6	67	Apixaban	10 (LMCA, LAD, and Cx: giant aneurysm)	Moderate size aneurysm	1103; age 69 y^b^	Missed diagnosis until MI at age 42 y

Abbreviations: CSA, cyclosporine A; CT, computed tomography; Cx, circumflex; DOAC, direct oral anticoagulant; IFX, infliximab; IVIG, intravenous immunoglobulin; KD, Kawasaki disease; LAD, left anterior descending coronary artery; LMCA, left main coronary artery; MI, myocardial infarction; ND, no data; RCA, right coronary artery.

^a^ Maximal dimension of aneurysm and calculated *Z*-score (internal dimension of aneurysm normalized for body surface area) are provided if data were available.

^b^ Treated with warfarin prior to starting DOAC.

^c^ KD-compatible illness not diagnosed as KD.

## Discussion

As a small case series, the generalizability of our findings to a larger KD population is unknown. The 3 adult patients with KD who experienced a major adverse cardiac event using 3 different DOACs demonstrate that these lesions were at substantial risk of thrombosis. In a multicenter, North American cohort of 80 patients with KD treated with warfarin, the mean cumulative incidence of coronary artery thrombosis was 6.7% over a median of 2.2 years.^5^ In a Japanese study of 273 patients with KD anticoagulated with warfarin for giant CAAs, the event-free survival at 10 years was only 52% for males and 75% for females.^6^ Thus, the long-term outlook for patients with giant CAA due to KD includes significant morbidity and mortality, which is not fully mitigated with systemic anticoagulation.

We recognize as limitations the case series design with no control group and only a single center experience. In conclusion, it is reasonable to consider a DOAC as chronic treatment for adult and pediatric patients with KD and giant CAAs. A large-scale, registry-based study of patients with KD and giant aneurysms using systemic anticoagulation could help to provide further guidance for optimal patient management.

## Notes

Supplement.
Data Sharing Statement
