# Supplementary material for: DOACs in Patients With Giant Coronary Artery Aneurysms After Kawasaki Disease
Source: JAMA Netw Open. 2023 Nov 10;6(11):e2343801. doi: 10.1001/jamanetworkopen.2023.43801 (PMC10638650; doi:10.1001/jamanetworkopen.2023.43801)
Supplement: Supplement. — Data Sharing Statement [file jamanetwopen-e2343801-s001.pdf]

Data Sharing Statement

Dummer. DOACs in Patients With Giant Coronary Artery Aneurysms After Kawasaki Disease. *JAMA Netw Open*. Published November 13, 2023. doi:10.1001/jamanetworkopen.2023.43801

Data
Data available: No

Additional Information
Explanation for why data not available: Data will not be available due to PHI issues.
